# Posttranscriptional regulation of Galectin-3 by miR-128 contributes to colorectal cancer progression

**DOI:** 10.18632/oncotarget.14839

**Published:** 2017-01-27

**Authors:** Weiqun Lu, Jia Wang, Guohua Yang, Nanrong Yu, Zhiliang Huang, Houwei Xu, Jianchang Li, Jiliang Qiu, Xiang Zeng, Shicai Chen, Nan Li, Haiying Liu

**Affiliations:** ^1^ Department of Gastrointestinal Neoplasms Surgery, Affiliated Cancer Hospital & Institute of Guangzhou Medical University, Guangzhou 510095, Guangdong, China

**Keywords:** colorectal cancer, Galectin-3, miR-128, cancer progression

## Abstract

Here we demonstrated that Galectin-3 protein level was frequently up-regulated in colorectal cancer (CRC) cells and tissues. Galectin-3 up-regulation correlated with CRC progression and predicted a shorter overall survival of CRC patients. Galectin-3 overexpression attenuated the chemo-sensitivity of cancer cells, but enhanced the potential invasiveness. To explore the mechanism for Galectin-3 dysregulation, we found that miR-128 level was frequently down-regulated in CRC and negatively correlated with Galectin-3 level. Using bioinformatics analysis and experimental validation, we showed that miR-128 could directly target Galectin-3 to repress its protein level. MiR-128 decrease associated with CRC progression and predicted a worse overall survival of CRC patients. Ectopic miR-128 expression enhanced the chemo-sensitivity of CRC cells *in vitro* and *in vivo*, and inhibited the potential invasiveness. Galectin-3 expression impaired the cancer suppressive effects of miR-128. These data highlighted the role of miR-128/Galectin-3 axis in colorectal cancer.

## INTRODUCTION

Colorectal cancer (CRC) is the third most common cancer and the third leading cause of cancer death in the United States, with an estimated 136,830 new cases and 50,310 deaths in 2014 [1]. Despite of early screening and development of new chemotherapeutic strategies, the CRC survival rates during the past 20 years have not substantially improved. The five-year survival rate for metastatic colon cancer is still less than 10%. Limited sensitivity to chemotherapy and metastasis are the most frequent reasons of treatment failure [2]. Thus, it is critical to understand the mechanisms for chemo-insensitivity and metastasis, and to identify potential therapeutic targets to improve patients’ outcomes.

Galectin-3 also known as Mac-2, CBP-30, hL-31, CBP-35, IgEBP and LBL, belongs to galectin family which is implicated in cell growth, differentiation, adhesion and malignant transformation. Galectin-3 exhibits pleiotropic biological and molecular functions via both extracellular and intracellular manners. Extracellularly, Galectin-3 interacts with cell surface and extracellular matrix glycoproteins and glycolipids to adjust microenvironment. Intracellularly, Galectin-3 interacts with cytoplasmic and nuclear proteins to modulate signaling pathways [3]. Galectins are often highly expressed in cancer cells and cancer-associated stromal cells, and their expression correlates with the aggressiveness of tumors and the acquisition of the metastatic phenotype, suggesting the involvement of galectins in tumor progression and disease outcome [4]. Inhibition of galectin-3 leads to loss of transformed phenotypes in breast cancer and thyroid papillary carcinoma cells. But overexpressed galectin-3 induces a transformed phenotype in normal thyroid follicular cell lines and causes human lymphoma Jurkat T cells to grow faster [5]. In regard to the mechanisms, Kloog et al. indicated that galectin-3 promotes the activation of RAF1 and PI3K signaling cascades but attenuates ERK activation [6]. Whereas, Wu et al. showed that galectin-3 enhances migration of colon cancer cells via activating K-Ras-Raf-Erk1/2 pathway [7]. Additionally, studies showed that galectin-3 binds to β-catenin and TCF4 and then activates wnt signaling targets genes such as cyclin D1 and c-Myc in breast cancer cells [8, 9]. Shi et al. also showed that inhibition of both wnt-2 and galectin-3 had synergistic effects on suppressing wnt signaling and inducing apoptosis [10]. Findings from Song et al. indicated that galectin-3 mediates nuclear β-catenin accumulation and wnt signaling in human colon cancer cells by regulation of glycogen synthase kinase-3beta activity [11]. Moreover, galectins may promote cancer cells resistance to platinum-based chemotherapy. Galectin-1 enhances chemo-resistance to cisplatin (cDDP) through the MAPK/COX-2 pathway in lung cancer [12]. Galectin-3 promotes chemo-resistance in prostate cancer, cholangiocarcinoma and thyroid carcinoma [13–15]. Nevertheless, the role and mechanisms of galectin-3 in chemo-sensitivity, invasion and metastasis in colon cancer remain elusive. Especially, the prognostic value and regulatory mechanisms of galectin-3 should be determined.

In the present study, we determined the expression pattern of galectin-3 in colon cancer cell lines and tissue samples, and systematically analyzed the relationships between galectin-3 expression level and clinicopathologic factors. We investigated the role of galectin-3 in chemo-sensitivity and invasion and identified miR-128 as a direct regulator of galectin-3 in cancer cells.

## RESULTS

### Galectin-3 is frequently overexpressed in CRC and predicts poor outcomes

**Figure 1:**
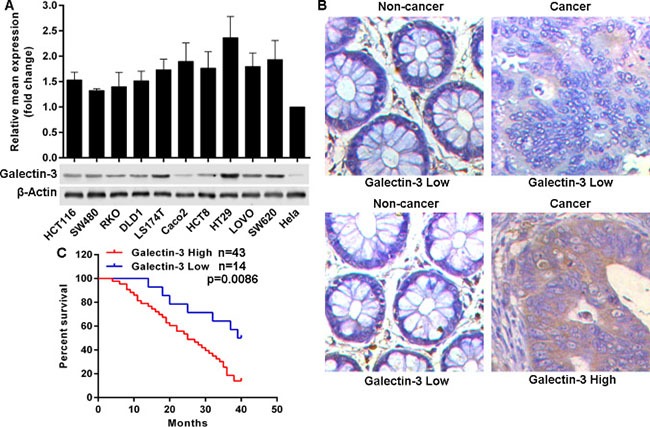
Galectin-3 is frequently overexpressed in CRC and predicts poor outcomes (**A**) Galectin-3 expression in mRNA and protein levels was determined in selected cell lines using qRT-PCR and western blot. (**B**) Reprehensive images of Galectin-3 protein level detected by immunohistochemical staining in 57 formalin-fixed paraffin-embedded CRC tissues, (20×). (**C**) Kaplan-Meier analysis estimated overall survival according to the Galectin-3 protein level.

To confirm the expression pattern of Galectin-3 in colon cancer, we initially evaluated the mRNA and protein levels in a series of colon rectal cancer (CRC) cell lines. We found that compared to levels in Hela cells, all ten CRC cell lines possessed relatively high protein level of Galectin-3, but not mRNA level (Figure 1A). Then we assessed Galectin-3 protein levels in 57 paraffin-embedded matched CRC and adjacent non-tumor colon tissues with follow-up data for patients. Results indicated that Galectin-3 was overexpressed in about 75.43% (43) CRC tissues (Figure 1B). Additionally, the relationship between Galectin-3 and clinicopathologic factors were analyzed using the Chi-Square test and results showed that Galectin-3 expression was significantly associated with differentiation status, Lymph node status and clinical stage, but was not significantly associated with gender, age, cancer origination, volume, and CEA levels at pre-operation (Table 1). Importantly, CRC patients with high Galectin-3 levels had poor overall survival times (Figure 1C). These results indicated that Galectin-3 was overexpressed in CRC, and was a potential poor prognostic marker for CRC patients.

**Table 1:** Correlation of Galectin-3 and miR-128 levels with clinicopathological parameters

Factor	Cases 57	Galectin-3 level		*P* value	miR-128 level		*P* value
		High 43 (75.43%)	Low 14 (24.57%)		High 20 (35.09%)	Low 37 (64.91%)	
Age, years				0.3187			0.5315
< 60	26	18	8		8	18	
≥ 60	31	25	6		12	19	
Gender				0.7062			0.6818
Male	35	27	8		13	22	
Female	22	16	6		7	15	
Tumor site				0.4636			0.3920
Colon	41	32	9		13	28	
Rectum	16	11	5		7	9	
Tumor size (CM)				0.0575			0.0656
< 5	17	10	7		9	8	
≥ 5	40	33	7		11	29	
Tumor differentiation				0.0422			0.0191
I	17	10	7		3	14	
II	24	22	2		7	17	
III	16	11	5		10	6	
Lymph node status				0.0152			0.0023
Negative	33	21	12		17	16	
Positive	24	22	2		3	21	
TNM stage				0.0014			0.0012
I + II	32	19	13		17	15	
III + IV	25	24	1		3	22	
CEA level (ng/ml)				0.4631			0.3093
< 5	12	8	4		6	6	
≥ 5	45	35	10		14	31	

### Galectin-3 attenuates chemo-sensitivity and promotes invasion in cancer cells

**Figure 2:**
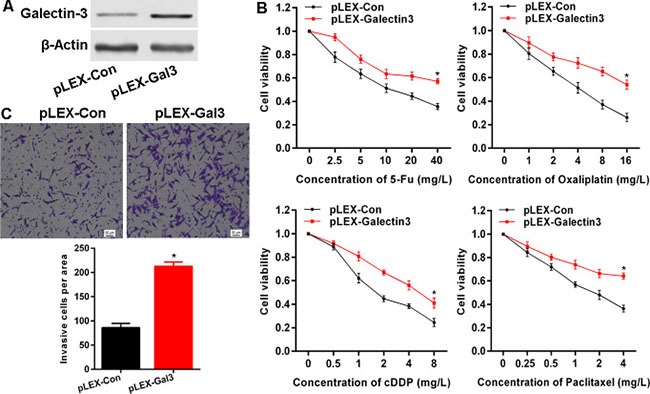
Galectin-3 attenuates chemo-sensitivity and promotes invasion in cancer cells (**A**) The protein level after transfection of Galetin-3 expressing plasmid was confirmed by western blot. (**B**) Galectin-3 overexpression attenuated chemo-sensitivity detected by MTS assay to determine proliferation. (**C**) Galectin-3 overexpression enhanced invasive potential examined using transwell assay. * vs control, *p* < 0.001.

Given the overexpression of Galectin-3 in CRC, it is necessary to elucidate the biological role in cancer cells. We sought to knockdown Galectin-3 with si-RNAs or sh-RNAs, but a series of designed or commercially obtained si-RNAs or sh-RNAs failed to knockdown Galectin-3 expression in CRC cell lines (data not shown). So here, we overexpressed Galectin-3 in Hela cells that have low endogenous Galectin-3 expression via transfection of Galectin-3 expressing plasmid pLEX-Galectin3 (Figure 2A), and found that overexpressed Galectin-3 markedly attenuated the sensitivity of Hela cells to 5-Fu, Oxaliplatin, cDDP and Paclitaxel (Figure 2B). Additionally, we investigated the effect of Galectin-3 on invasion and demonstrated that overexpressed Galectin-3 remarkably enhanced the invasion potential of Hela cells (Figure 2C).

### Galectin-3 levels are inversely correlated with miR-128 expression in CRC

**Figure 3:**
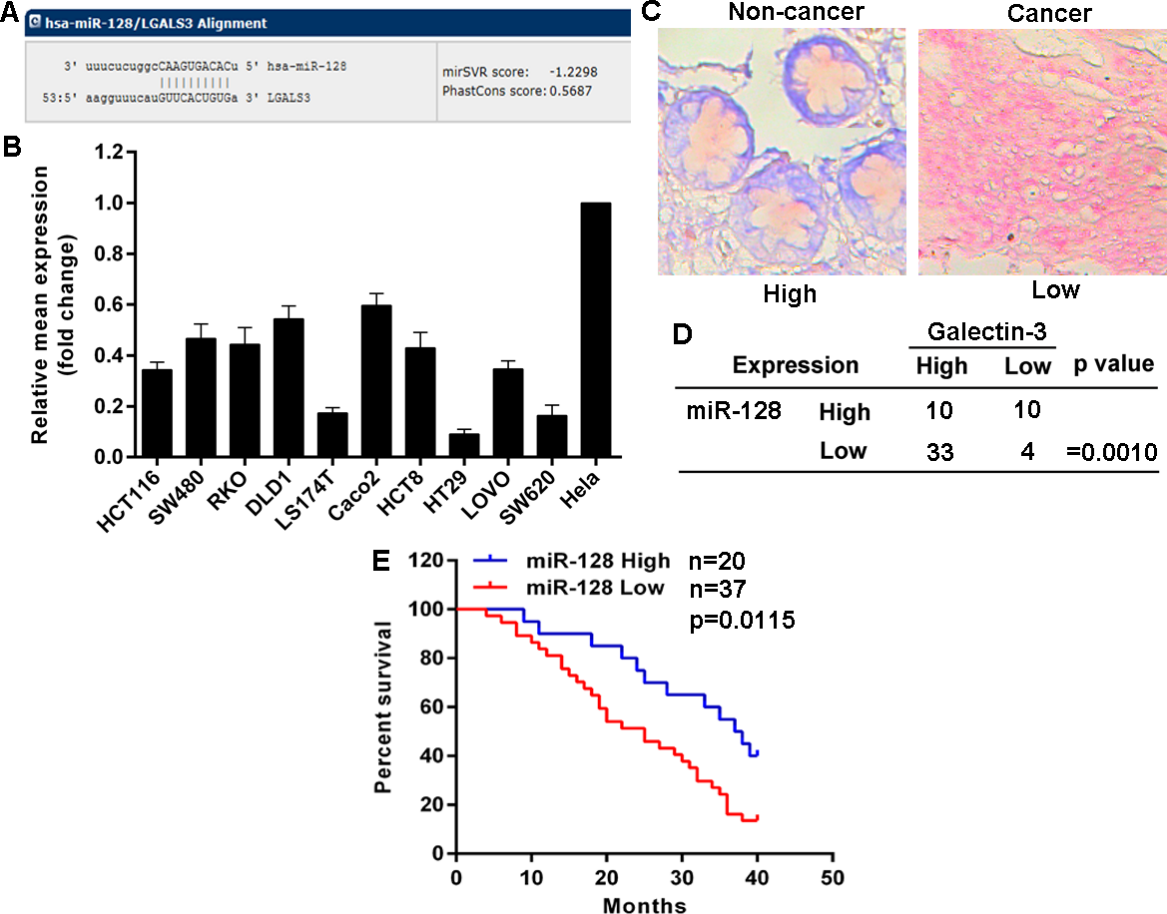
Galectin-3 levels are inversely correlated with miR-128 expression in CRC (**A**) Schematic of the putative binding site of miR-128 in 3′-UTR of Galectin-3 after prediction using the public database (http://www.mirbase.org). (**B**) Relative miR-222 expression was determined by qRT-PCR. (**C**) Representative images of miR-128 expression detected by ISH (red staining as nucleus and blue staining as miR-128 level). (**D**) Correlation between miR-128 level and Galectin-3 protein level in CRC tissues determined by a χ^2^ test. (**E**) Kaplan-Meier analysis estimated overall survival according to the miR-128 level.

Given that there are no effective si-RNAs or sh-RNAs to knockdown Galectin-3 expression and that Galectin-3 is overexpressed and plays potential role in cancer cells, to explore mechanisms for Galectin-3 up-regulation, we focused on miRNAs which have been importantly involved in many types of cancers. Potential miRNAs were predicted using the public database (http://www.mirbase.org) and miR-128 with a binding site in the 3’-UTR of Galectin-3 mRNA was selected for further expression and function confirmation (Figure 3A). Expectedly, the detection of miR-128 expression in above cell lines showed that miR-128 expression in CRC cell lines was much lower than that in Hela cells with low endogenous Galectin-3 protein level (Figure 3B). Additionally, miR-128 expression in above CRC tissues was detected using miRNAs specific *in situ* hybridizations (ISH). We found miR-128 was down-regulated in 37 (64.91%) CRC tissues compared to matched non-cancer tissues (Figure 3C). 33 out of the 37 tissues possessed high Galectin-3 protein level, but only 10 out of the 20 tissues with high miR-128 expression possessed high Galectin-3 protein level. To address whether miR-128 and Galectin-3 have potential in-parallel relationship, we analyzed miR-128 and Galectin-3 expression using Pearson correlation coefficient and found that Galectin-3 up-regulation was accompanied with miR-128 down-regulation, indicating a negative correlation between miR-128 and Galectin-3 expression (Figure 3D). Importantly, miR-128 decrease was significantly associated with differentiation status, Lymph node status and clinical stage (Table 1). CRC patients with low miR-128 levels had shorter overall survival times (Figure 3E). These findings indicated a potential role of miR-128/Galectin-3 axis in CRC.

### MiR-128 targets Galectin-3 in cancer cells

**Figure 4:**
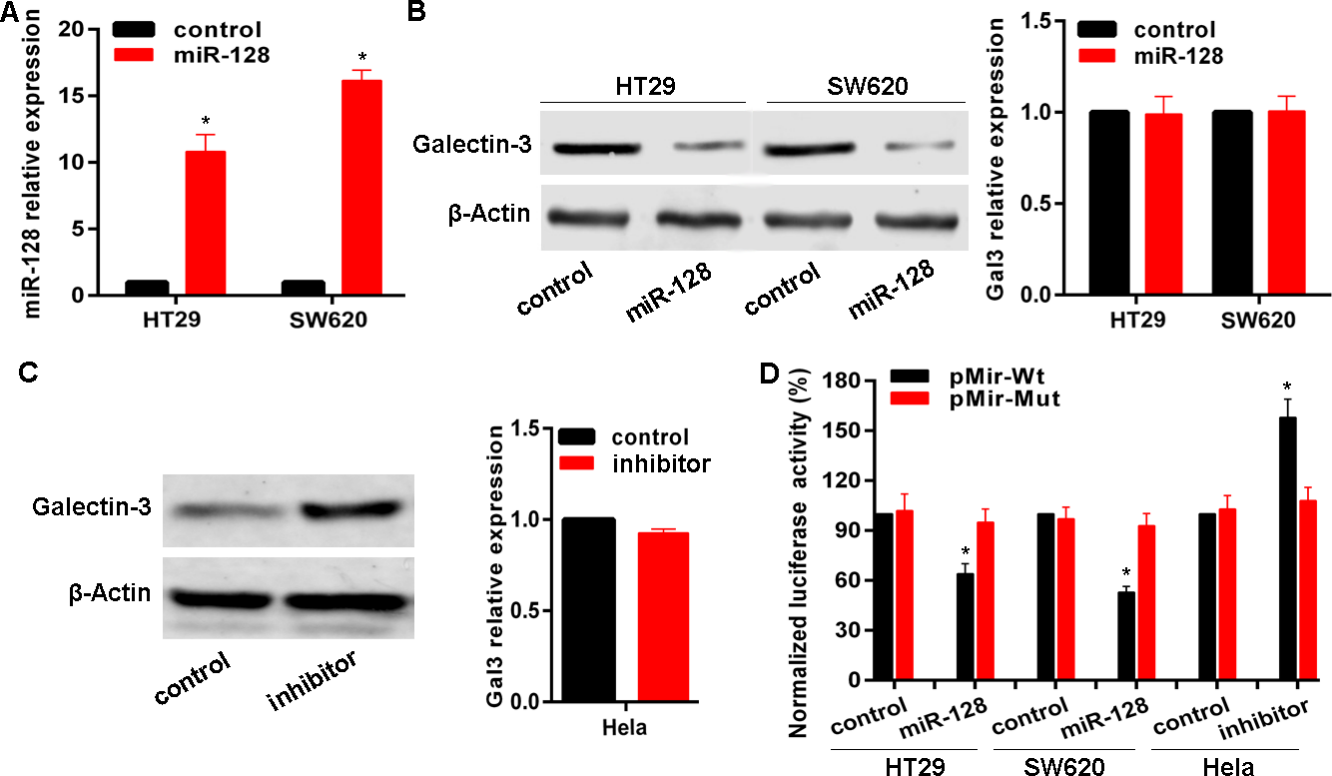
miR-128 targets Galectin-3 in cancer cells (**A**) The miR-128 level after transfection of miR-128 expressing plasmid HIVH1-miR-128 was confirmed by qRT-PCR. (**B**, **C**) Galectin-3 protein and mRNA levels after transfection were detected with miR-128 expressing plasmid or inhibitor. (**D**) Luciferase reporter assay in cell lines cotransfected with miR-128 expressing plasmid or miR-128 inhibitor, a luciferase reporter containing wild-type Galectin-3 3′-UTR or a mutant version, and a renilla luciferase reporter for normalization. The mean of the results from cells transfected with pMir-Wt and control was set as 100. * vs related control, *p* < 0.01.

Given the inverse correlation between miR-128 and Galectin-3 in cancer cells, and tissues combined with bioinformatics analysis, we posited that miR-128 potentially regulated Galectin-3 expression. To this end, we transfected HT29 and SW620 cells with miR-128 expressing plasmid HIVH1-miR-128 to establish stable cell lines (Figure 4A). Results indicated that miR-128 successfully down-regulated Galectin-3 protein levels in HT29 and SW620 cell lines, but not mRNA levels (Figure 4B). In contrast, transfection with miR-128 inhibitor up-regulated Galectin-3 protein level in Hela cells rather than mRNA level (Figure 4C). To determine whether miR-128 represses Galectin-3 expression through a process triggered by the interaction between miR-128 and Galectin-3 3’-UTR region, we constructed a reporter plasmid containing firefly luciferase fused with Galectin-3 3’-UTR region containing miR-128 binding site and cotransfected the reporter plasmid with miR-128 expressing plasmid or control in HT-29 or SW620 cell lines. As shown, miR-128 overexpression resulted into a remarkable decrease of luciferase activity driven by Galectin-3 3’-UTR in HT-29 and SW620 cell lines (Figure 4D). But cotransfection performed in Hela cells indicated that miR-128 inhibitor enhanced the luciferase activity (Figure 4D). These results strongly demonstrated the specificity of miR-128 targeting *Galectin-3* mRNA.

### MiR-128 sensitizes CRC cells to chemotherapy and inhibits invasion

**Figure 5:**
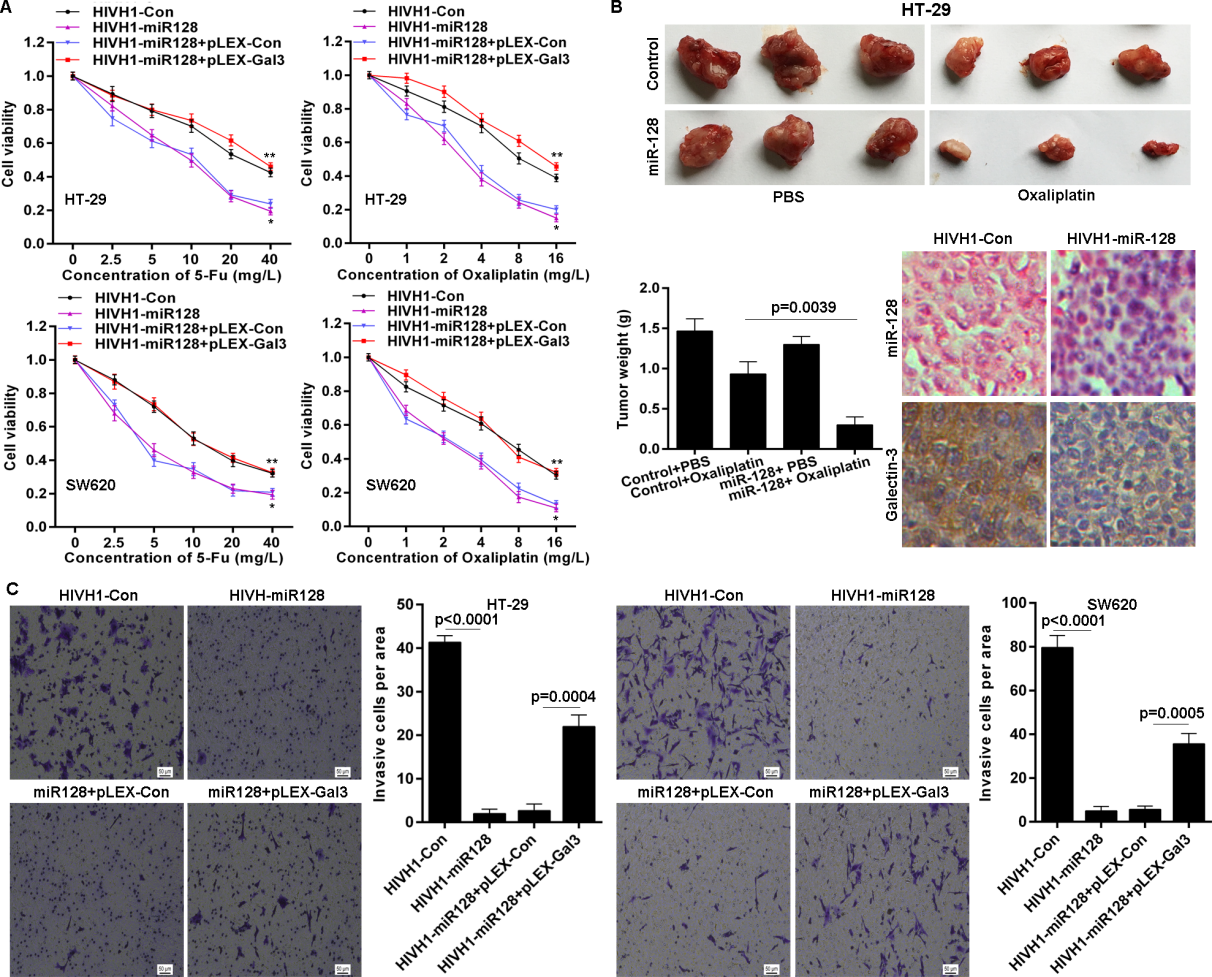
miR-128 sensitizes CRC cells to chemotherapy and inhibits invasion (**A**) miR-128 overexpression enhanced chemo-sensitivity detected by MTS assay but Galectin-3 overexpression abolished the effect of miR-128. * vs HIVH1-Con, *p* < 0.001; ** vs HIVH1-miR128+pLEX-Con, *p* < 0.001. (**B**) The chemo-sensitivity *in vivo* was monitored after miR-128 overexpression in HT-29 cell line. The tumor wet weights were recorded. The miR-128 and Galectin-3 levels in xenograft tissues were detected by ISH and immunohistochemical staining, respectively. (**C**) miR-128 inhibited the invasive potential of CRC cell lines, but Galectin-3 impaired the effect of miR-128, determined by transwell assay.

Given the above observations that miR-128 targets Galectin-3 and that Galectin-3 is involved in chemo-sensitivity and invasion, we used a gain- or loss-of-function approach to investigate the biological role of miR-128. We found that miR-128 overexpression significantly sensitized HT-29 and SW620 CRC cells to chemotherapy induced growth inhibition (Figure 5A). Due to down-regulation of miR-128 in CRC cells and Galectin-3 is a direct target of miR-128, it is necessary to investigate whether miR-128 exerts its role via suppressing Galectin-3 expression. Here, HT-29 and SW620 cell lines with stable overexpression of miR-128, were transfected with Galectin-3 expressing plasmid pLEX-Galectin3 without 3’-UTR. We found that ectopic Galectin-3 expression significantly abolished miR-128 mediated chemo-sensitization (Figure 5A). Then, we tested whether miR-128 could play a role in chemotherapy using nude mice xenograft model. We injected subcutaneously into the oxter of athymic mice with miR-128 overexpressed HT-29 cells and their related control cells. After 12 days, these mice were treated with PBS (control) or Oxaliplatin (5 mg/kg) every 3 days for seven cycles. We found that miR-128 slightly influenced tumor growth, but markedly affected the chemo-sensitivity *in vivo*. The tumor weight of miR-128 overexpressed xenografts decreased to greater extent than that of control xenografts, in response to Oxaliplatin treatment (Figure 5B). We also found that miR-128 was exactly overexpressed in xenografts and that miR-128 repressed Galectin-3 protein level *in vivo* (Figure 5B). In addition, we determined the role of miR-128 on invasion. It is indicated that overexpressed miR-128 inhibited invasion potential of HT-29 cells and SW620 cells (Figure 5C). However, ectopic expressed Galectin-3 obviously impaired miR-128 induced invasion inhibition in HT-29 and SW620 cell lines (Figure 5C). These results imply that miR-128 is involved in chemo-sensitivity and invasion, but Galectin-3 impairs the effects of miR-128 in CRC cells.

## DISCUSSION

Limited sensitivity to chemotherapy, invasion and metastasis mostly results into cancer progression and treatment failure for human colorectal cancer (CRC). Galectin-3 overexpression has been involved in several human cancer types [4]. Here, we investigated the expression pattern, function and clinical significance of Galectin-3 in CRC. Our findings indicated that Galectin-3 protein level was frequently highly expressed in CRC tissues and cells, and correlated with CRC progression. High Galectin-3 protein level maybe resulted from post-transcriptional regulation. In order to investigate the effects of Galectin-3 on CRC cells biological behaviors, we firstly tried to knockdown Galectin-3 expression using sh-RNAs or si-RNAs, but neither all commercially obtained sh-RNAs nor si-RNAs could successfully knockdown Galectin-3 expression in CRC cell lines. We performed gain-of-function assay in Hela cells and found that Galectin-3 overexpression enhanced chemo-resistance. Thus, we will employ the CRISPR/Cas9 system to knockout Galetcin-3 to explore its functions in our further studies.

Because exogenous sh-RNAs or si-RNAs failed to knockdown Galectin-3 expression, here we sought endogenous miRNAs that would regulate Galetin-3 expression. Coincidentally, differential Galectin-3 expression presented on protein level but not on mRNA level. Bioinformatics analysis implied that miR-128 would target Galectin-3. Subsequent experimental data indicated that miR-128 level was inversely correlated with Galectin-3 protein level in CRC tissues and that Galectin-3 was a direct target of miR-128. miRNAs are a class of endogenous small non-coding RNAs that post-transcriptionally regulate genes expression by targeting the 3′-untranslated region (3′-UTR) of target mRNAs through either translational repression or mRNA degradation [16]. miRNAs deregulation have been reported to be involved in a wide range of physiological and pathological processes, such as cell proliferation, development, differentiation, carcinogenesis and metastasis [17]. Aberrant expression of miR-128 has been found to occur in a variety of human cancer types and functions as a key regulator of oncogenic properties. MiR-128 is highly expressed in normal brain tissues but the miR-128 level significantly decreases in glioma tissues. As an tumor suppressor in glioma, miR-128 inhibits glioma cells proliferation and self-renewal via targeting BMI1 [18], E2F3a [19] and mitogenic kinases [20]. MiR-128 reduce was also found in serum from glioma patients and could be used as a potential biomarker for glioma [21]. Up-regulation of miR-128 inhibited Reelin and DCX expression and then reduced neuroblastoma cell notility and invasiveness [22]. Moreover, miR-128 has been shown to regulate differentiation in hematopoietic stem progenitor cells [23] and muscle-side population cells [24]. Here, our data demonstrated that miR-128 was frequently down-regulated in CRC. Restored miR-128 expression sensitized CRC cells to chemotherapy *in vitro* and *in vivo*. MiR-128 also inhibited the potential invasiveness of CRC cells. But, Galectin-3 expression markedly reversed the effects of miR-128 as a cancer suppressor in CRC cells. However, whether miR-128 could serve as a serum biomarker for CRC and the mechanisms for abnormal expression of miR-128 will be elucidated in our further studies.

Collectively, in our present study, we extend the knowledge on Galectin-3 by highlighting its role in colorectal cancer progression. Our findings suggest miR-128/Galectin-3 axis can be a biomarker for colorectal cancer and a candidate molecular target to improve the efficacy of colorectal cancer treatment.

## MATERIALS AND METHODS

### Cell lines and tissue specimens

The human CRC cell lines HCT116, SW480, RKO, DLD1, LS174T, Caco2, HCT8, HT29, LOVO and SW620, and the Hela cell lines were obtained from the Affiliated Cancer Hospital & Institute of Guangzhou Medical University (Guangzhou, China). Above cell lines were cultured in RPMI-1640 (Gibco, Carlsbad, CA, USA) with 10% inactivated fetal bovine serum (Gibco) and 100 units/ml penicillin and 100 μg/ml streptomycin at 37°C in a humidified atmosphere containing 5% CO_2_. Fifty-seven CRC tissue specimens were obtained from patients at the Affiliated Cancer Hospital & Institute of Guangzhou Medical University. Overall survival was computed from the day of surgery to the day of death or of last follow-up. The study was approved by the ethics committee of the Affiliated Cancer Hospital & Institute of Guangzhou Medical University. The 5-Fu, Oxaliplatin, cDDP and Paclitaxel were from Sigma- Aldrich (Steinheim, Germany).

### Real-time PCR for mRNA and miRNAs

The mRNAs and miRNAs were extracted simultaneously were isolated and purified with miRNA isolation system (OMEGA Bio-Tek, Norcross, GA, USA). For mRNA qRT-PCR, cDNAs from the mRNAs were synthesized with the first-strand synthesis system (Thermo Scientific, Glen Brunie, MA, USA). Real-time PCR was carried out according to standard protocols using an ABI 7500 with SYBR Green detection (Applied Biosystems, Foster City, CA, USA). GAPDH was used as an internal control and the qRT-PCR was repeated three times. The primers for GAPDH were: forward primer 5′-ATTCCATGGCACCGTCAAGGCTGA-3′, reverse primer 5′-TTCTCCATGGTGGTGAAGACGCCA-3′; primers for Galectin-3 were: forward primer 5′- ATGGCAGACAATTTTTCGCTCC-3′, reverse primer 5′-GCCTGTCCAGGATAAGCCC-3′. For miRNA qRT-PCR, cDNA was generated with the miScript II RT Kit (QIAGEN, Hilden, Germany) and the quantitative real-time PCR (qRT-PCR) was done by using the miScript SYBR Green PCR Kit (QIAGEN) following the manufacturer's instructions. The miRNA sequence-specific qRT-PCR primers for miR-128 and endogenous control RNU6 were purchased from QIAGEN, and the qRT-PCR analysis was carried out using 7500 Real-Time PCR System (Applied Biosystems). The gene expression threshold cycle (CT) values of miRNAs were calculated by normalizing with internal control RNU6 and relative quantization values were calculated.

### Western blot

Total proteins were extracted from corresponding cells using the RIPA buffer (Thermo Scientific) in the presence of Protease Inhibitor Cocktail (Thermo Scientific). The protein concentration of the lysates was measured using a BCA Protein Assay Kit (Thermo Scientific). Equivalent amounts of protein were resolved and mixed with 5×Lane Marker Reducing Sample Buffer (Thermo Scientific), electrophoresed in a 10% SDS–acrylamide gel and transferred onto Immobilon-P Transfer Membrane (Millipore, Billerica, MA, USA). The membranes were blocked with 5% non-fat milk in Tris-buffered saline and then incubated with primary antibodies followed by secondary antibody. The signal was detected using an ECL detection system (Millipore). The Galectin-3 antibody was from Santa Cruz Biotechnology (Dallas, Texas, USA). The β-Actin antibody was from Cell Signaling Technology (Danvers, MA, USA). HRP-conjugated secondary antibody was from Thermo.

### Immunohistochemistry

The sections were dried at 55°C for 2 h and then deparaffinized in xylene and rehydrated using a series of graded alcohol washes. The tissue slides were then treated with 3% hydrogen peroxide in methanol for 15 min to quench endogenous peroxidase activity and antigen retrieval then performed by incubation in 0.01 M sodium cirate buffer (pH 6.0) and heating using a microwave oven. After a 1 h preincubation in 10% goat serum, the specimens were incubated with primary antibody overnight at 4°C. The tissue slides were treated with a non-biotin horseradish peroxidase detection system according to the manufacturer's instruction (DAKO, Glostrup, Denmark). Two different pathologists evaluated the immunohistological samples. The intensity of immunostaining was taken into consideration when analyzing the data. The intensity of staining was scored from 0 to 3 and the expression was classified as high if the score was ≥ 2, and as low if the score was ≤ 1.

### Cells transfection

Cells were trypsinized, counted and seeded into six-well plates the day before transfection to ensure 70% cell confluency on the day of transfection. The transfection of the Galectin-3 expressing plasmid pLEX-Galectin-3, miR-128 expressing plasmid HIVH1-miR-128 and related controls was carried out using Lipofectamine 2000 (Invitrogen, Carlsbad, CA, USA) in accordance with the manufacturer's instructions. To establish stable cell lines, the puromycin was sued for selection. The miR-128 inhibitor and relative control were purchased from Ambion, and were transfected with Lipofectamine 2000. The inhibitor and controls were used at a final concentration of 100 nM. At 48 h post-transfection, follow-up experiments were performed.

### Cell invasion assay

Invasion of cells was assessed using the Cell Invasion Assay Kit (BD Biosciences, Franklin Lakes, NJ, USA) according to the manufacturer's instructions. Briefly, at 36 h post-transfection, 3 × 10^4^ cells in 300 μl serum-free medium were added to the upper chamber precoated with ECMatrix™ gel. Then, 0.5 ml of 10% FBS-containing medium was added to the lower chamber as a chemoattractant. Cells were incubated for 24 h at 37°C, and then non-invading cells were removed with cotton swabs. Cells that migrated to the bottom of the membrane were fixed with pre-cold methanol and stained with 2% Giemsa solution. Stained cells were visualized under a microscope.

### MTS assay

The CellTiter 96 AQueous One Solution Cell Proliferation Assay kit (Promega, Madison, WI, USA) was used to determine the sensitivity of cells to 5-Fu, Oxaliplatin, cDDP and Paclitaxel. Briefly, cells were seeded in 96-well plates at a density of 4 × 10^3^ cells/well (0.2 ml/well) for 24h before use. The culture medium was replaced with fresh medium containing 5-Fu, Oxaliplatin, cDDP or Paclitaxel at different concentrations and cells were then incubated for a further 48 hours. Then, MTS (0.02 ml/well) was added. After a further 2 hours incubation, the absorbance at 490 nm was recorded for each well on the BioTek Synergy 2. The absorbance represented the cell number and was used for the plotting of dose-cell number curves.

### miRNA luciferase reporter assay

Two single strands of the wild type 3’UTR with miR-128 binding site (as wild type) and two single strands of the mutant type with 10 bases deletion in the miR-128 binding site (as mutant type), of Galectin-3 were synthesized with restriction sites for SpeI and HindIII located at both ends of the oligonucleotides for further cloning. The corresponding sense and antisense strands were annealed and subsequently cloned into pMir-Report plasmid downstream of firefly luciferase reporter gene. Cells were seeded in 96 well-plates and co-transfected with the wild type or mutant type pMir-Report luciferase vector, pRL-TK Renilla luciferase vector and miR-128 expressing plasmid or miR-128 inhibitor. 48h after cotransfection, the luciferase activities were determined using a Dual-Luciferase Reporter Assay System (Promega) where the Renilla luciferase activity was used as internal control and the firefly luciferase activity was calculated as the mean ± SD after being normalized by Renilla luciferase activity.

### Xenograft model in nude mice

Xenograft tumours were generated by subcutaneous injection of miR-128 overexpressing or control HT-29 cells (2 × 10^6^) respectively, into the oxter of 4–6 week-old Balb/C athymic nude mice. All mice were housed and maintained under specific pathogen-free conditions, and all experiments were approved by the Use Committee for Animal Care and performed in accordance with institutional guidelines. 12 days after cancer cell transplantation, the mice were injected intraperitoneally with Oxaliplatin (5 mg/kg). The treatment was administered every 3 days for seven cycles. Tumors were harvested and weighed at the experimental endpoint.

### miRNA In Site hybridizations (ISH) assay

The miR-128 expression in CRC samples was detected by In Site Hybridizations (ISH) with kit from Exiqon (Vedbaek, Denmark) according to the manufacturer's instructions. Briefly, the sections were dried at 65°C for 3 h and then deparaffinized in xylene and ethanol at room temperature (RT) followed with a 10 min incubation with proteinase-k at 37°C. After dehydration in ethanol, sections were hybridizated with 40 nM double-DIG LNA^™^ miR-128 probe 55°C for 1 h. After wash in SSC buffer at hybridization temperature and incubation with blocking solution for 15 min, the anti-DIG reagent sheep anti-DIG-AP (Roche, Mannheim, Germany) was applied and incubated for 60 min at RT. After wash in PBST, the sections were incubated with AP substrate NBT-BCIP (Roche) for 2 h at 30°C and incubated in KTBT buffer to stop reaction. Then the nuclear counter stain Nuclear Fast Red™ (Vector labs, Burlingame, CA) was applied for 1 min for nuclear counter staining, and slides were rinsed in tap water for 10 min. after dehydrated in ethanol and mounted, the sections were investigated and analyzed under microcopy.

### Statistical analysis

All data are expressed as means ± standard deviation from three independent experiments. Statistical analyses were performed using SPSS16.0 software (SPSS, Chicago, IL). The differences between groups were analyzed using Student's *t*-test. Pearson's correlation analysis was used to determine the correlation between miR-128 expression and Galetin-3 protein level in the 57 tissues. Survival curves were constructed using the Kaplan–Meier method and analyzed by the log-rank test. *P* values less than 0.05 were considered statistically significant.

## References

[1] Siegel R, Desantis C, Jemal A (2014). Colorectal cancer statistics, 2014. CA Cancer J Clin.

[2] Wang H, Zhang L, Yang X, Jin Y, Pei S, Zhang D, Zhang H, Zhou B, Zhang Y, Lin D (2015). PUMA mediates the combinational therapy of 5-FU and NVP-BEZ235 in colon cancer. Oncotarget.

[3] Liu FT, Rabinovich GA (2005). Galectins as modulators of tumour progression. Nat Rev Cancer.

[4] Chung LY, Tang SJ, Wu YC, Sun GH, Liu HY, Sun KH (2015). Galectin-3 augments tumor initiating property and tumorigenicity of lung cancer through interaction with β-catenin. Oncotarget.

[5] Yoshii T, Inohara H, Takenaka Y, Honjo Y, Akahani S, Nomura T, Raz A, Kubo T (2001). Galectin-3 maintains the transformed phenotype of thyroid papillary carcinoma cells. Int J Oncol.

[6] Elad-Sfadia G, Haklai R, Balan E, Kloog Y (2004). Galectin-3 augments K-Ras activation and triggers a Ras signal that attenuates ERK but not phosphoinositide 3-kinase activity. J Biol Chem.

[7] Wu KL, Huang EY, Jhu EW, Huang YH, Su WH, Chuang PC, Yang KD (2013). Overexpression of galectin-3 enhances migration of colon cancer cells related to activation of the K-Ras-Raf-Erk1/2 pathway. J Gastroenterol.

[8] Shimura T, Takenaka Y, Tsutsumi S, Hogan V, Kikuchi A, Raz A (2004). Galectin-3, a novel binding partner of beta-catenin. Cancer Res.

[9] Shimura T, Takenaka Y, Fukumori T, Tsutsumi S, Okada K, Hogan V, Kikuchi A, Kuwano H, Raz A (2005). Implication of galectin-3 in Wnt signaling. Cancer Res.

[10] Shi Y, He B, Kuchenbecker KM, You L, Xu Z, Mikami I, Yagui-Beltran A, Clement G, Lin YC, Okamoto J, Bravo DT, Jablons DM (2007). Inhibition of Wnt-2 and galectin-3 synergistically destabilizes beta-catenin and induces apoptosis in human colorectal cancer cells. Int J Cancer.

[11] Song S, Mazurek N, Liu C, Sun Y, Ding QQ, Liu K, Hung MC, Bresalier RS (2009). Galectin-3 mediates nuclear beta-catenin accumulation and Wnt signaling in human colon cancer cells by regulation of glycogen synthase kinase-3beta activity. Cancer Res.

[12] Chung LY, Tang SJ, Sun GH, Chou TY, Yeh TS, Yu SL, Sun KH (2012). Galectin-1 promotes lung cancer progression and chemoresistance by upregulating p38 MAPK, ERK, and cyclooxygenase-2. Clin Cancer Res.

[13] Wang Y, Nangia-Makker P, Balan V, Hogan V, Raz A (2010). Calpain activation through galectin-3 inhibition sensitizes prostate cancer cells to cisplatin treatment. Cell Death Dis.

[14] Wongkham S, Junking M, Wongkham C, Sripa B, Chur-In S, Araki N (2009). Suppression of galectin-3 expression enhances apoptosis and chemosensitivity in liver fluke-associated cholangiocarcinoma. Cancer Sci.

[15] Lavra L, Ulivieri A, Rinaldo C, Dominici R, Volante M, Luciani E, Bartolazzi A, Frasca F, Soddu S, Sciacchitano S (2009). Gal-3 is stimulated by gain-of-function p53 mutations and modulates chemoresistance in anaplastic thyroid carcinomas. J Pathol.

[16] Zheng G, Li N, Jia X, Peng C, Luo L, Deng Y, Yin J, Song Y, Liu H, Lu M, Zhang Z, Gu Y, He Z (2016). MYCN-mediated miR-21 overexpression enhances chemo-resistance via targeting CADM1 in tongue cancer. J Mol Med (Berl).

[17] Pasquinelli AE (2012). MicroRNAs and their targets: recognition, regulation and an emerging reciprocal relationship. Nat Rev Genet.

[18] Godlewski J, Nowicki MO, Bronisz A, Williams S, Otsuki A, Nuovo G, Raychaudhury A, Newton HB, Chiocca EA, Lawler S (2008). Targeting of the Bmi-1 oncogene/stem cell renewal factor by microRNA-128 inhibits glioma proliferation and self-renewal. Cancer Res.

[19] Zhang Y, Chao T, Li R, Liu W, Chen Y, Yan X, Gong Y, Yin B, Liu W, Qiang B, Zhao J, Yuan J, Peng X (2009). MicroRNA-128 inhibits glioma cells proliferation by targeting transcription factor E2F3a. J Mol Med (Berl).

[20] Papagiannakopoulos T, Friedmann-Morvinski D, Neveu P, Dugas JC, Gill RM, Huillard E, Liu C, Zong H, Rowitch DH, Barres BA, Verma IM, Kosik KS (2012). Pro-neural miR-128 is a glioma tumor suppressor that targets mitogenic kinases. Oncogene.

[21] Sun J, Liao K, Wu X, Huang J, Zhang S, Lu X (2015). Serum microRNA-128 as a biomarker for diagnosis of glioma. Int J Clin Exp Med.

[22] Evangelisti C, Florian MC, Massimi I, Dominici C, Giannini G, Galardi S, Buè MC, Massalini S, McDowell HP, Messi E, Gulino A, Farace MG, Ciafrè SA (2009). MiR-128 up-regulation inhibits Reelin and DCX expression and reduces neuroblastoma cell motility and invasiveness. FASEB J.

[23] Georgantas RW, Hildreth R, Morisot S, Alder J, Liu CG, Heimfeld S, Calin GA, Croce CM, Civin CI (2007). CD34+ hematopoietic stem-progenitor cell microRNA expression and function: a circuit diagram of differentiation control. Proc Natl Acad Sci USA.

[24] Motohashi N, Alexander MS, Casar JC, Kunkel LM (2012). Identification of a novel microRNA that regulates the proliferation and differentiation in muscle side population cells. Stem Cells Dev.

