# Correction: Posttranscriptional regulation of Galectin-3 by miR-128 contributes to colorectal cancer progression

**DOI:** 10.18632/oncotarget.24558

**Published:** 2018-02-23

**Authors:** Weiqun Lu, Jia Wang, Guohua Yang, Nanrong Yu, Zhiliang Huang, Houwei Xu, Jianchang Li, Jiliang Qiu, Xiang Zeng, Shicai Chen, Nan Li, Haiying Liu

**Affiliations:** ^1^ Department of Gastrointestinal Neoplasms Surgery, Affiliated Cancer Hospital & Institute of Guangzhou Medical University, Guangzhou 510095, Guangdong, China

**This article has been corrected:** The updated Acknowledgments and Funding information is provided below:

**ACKNOWLEDGMENTS AND FUNDING**

This study was supported by grants from the National Natural Science Foundation of China (No. 81401989). Guangzhou Science and Technology Plan Projects (Health Medical Collaborative Innovation Program of Guangzhou) (No. 201400000001-4).

Original article: Oncotarget. 2017; 8:15242-15251. https://doi.org/10.18632/oncotarget.14839

